# High Cognitive Load Situations Decrease Both Gait and Cognitive Performance

**DOI:** 10.1093/geroni/igaa057.958

**Published:** 2020-12-16

**Authors:** Hyeon Jung Kim, Farahnaz Fallahtafti, Jennifer Yentes, Dawn Venema, Julie Blaskewicz Boron

**Affiliations:** University of Nebraska Omaha, Omaha, Nebraska, United States

## Abstract

A high cognitive load situation (HCLS) is completing two or more tasks simultaneously (i.e. walking while talking). Differential allocation of attentional demands creates HCLS, potentially deteriorating cognitive and/or gait performance, impacting fall risk. This study investigated whether different load types [(Single-task (ST): talking/walking only, and HCLS: walking while talking on a phone)] impacted gait and cognitive performance among young (n=8; age=23.16±1.96yrs), middle-aged (n=14; age=44.79±7.42yrs), and older (n=15; age=74.47±3.91yrs) adults. In 3-minute trials, participants completed single-task walking (ST-W) and phone conversations with easy (e.g., favorite food, ST-E) and difficult (e.g., personal relationships, ST-D) topics, and also combined walking and talking (easy: HCLS-E and difficult: HCLS-D). For gait, speed, step length (SL) and stride width (SW) were analyzed with 3(ST-W, HCLS-E, HCLS-D) x 3(Age) repeated-measures ANOVAs. HCLS resulted in slower speed (p <.001, shorter SL (p <.001), and wider SW (p=.008) across groups. Older adults exhibited shorter SL across walking conditions (p=.002) compared to young and middle-aged. For cognition, Word Count (WC) and Authenticity (i.e. honesty) were analyzed with 2(Evs.D) x 2(STvs.HCLS) x 3(Age) repeated-measures ANOVAs. Main effects emerged for conversation topic in WC (p=.04) and Authenticity (p<.001); difficult topics negatively impacted participants’ cognitive performance, likely resulting from higher attention to maintain conversations without personal interactions (i.e. visual cues). Marginal age-group differences (p=.056) revealed older age resulted in less authentic conversations. The HCLS in this study negatively impacted gait and cognitive performance. Understanding this relationship may ultimately inform development of interventions to improve allocation of attentional demands, potentially mitigating fall risk.

